# Low Ligation Plus High Dissection *Versus* High Ligation of the Inferior Mesenteric Artery in Sigmoid Colon and Rectal Cancer Surgery: A Meta-Analysis

**DOI:** 10.3389/fonc.2021.774782

**Published:** 2021-11-11

**Authors:** Tzu-Chieh Yin, Yen-Cheng Chen, Wei-Chih Su, Po-Jung Chen, Tsung-Kun Chang, Ching-Wen Huang, Hsiang-Lin Tsai, Jaw-Yuan Wang

**Affiliations:** ^1^ Division of General and Digestive Surgery, Department of Surgery, Kaohsiung Medical University Hospital, Kaohsiung Medical University, Kaohsiung, Taiwan; ^2^ Department of Surgery, Kaohsiung Municipal Tatung Hospital, Kaohsiung Medical University, Kaohsiung, Taiwan; ^3^ Division of Colorectal Surgery, Department of Surgery, Kaohsiung Medical University Hospital, Kaohsiung Medical University, Kaohsiung, Taiwan; ^4^ Graduate Institute of Clinical Medicine, College of Medicine, Kaohsiung Medical University, Kaohsiung, Taiwan; ^5^ Department of Surgery, Faculty of Medicine, College of Medicine, Kaohsiung Medical University, Kaohsiung, Taiwan; ^6^ Graduate Institute of Medicine, College of Medicine, Kaohsiung Medical University, Kaohsiung, Taiwan; ^7^ Center for Cancer Research, Kaohsiung Medical University, Kaohsiung, Taiwan; ^8^ Center for Liquid Biopsy and Cohort Research, Kaohsiung Medical University, Kaohsiung, Taiwan

**Keywords:** high ligation, low ligation with high dissection, sigmoid colon cancer, rectal cancer (RC), inferior mesenteric artery (IMA), left colic artery

## Abstract

**Background:**

Whether high or low ligation of the inferior mesenteric artery (IMA) is superior in surgery for rectal and sigmoid colon cancers remains controversial. Although several meta-analyses have been conducted, the level of lymph node clearance was poorly defined. We performed a meta-analysis comparing high and low ligation of the IMA for sigmoid colon and rectal cancers, with emphasis on high dissection of the lymph node at the IMA root in all the included studies.

**Methods:**

PubMed, MEDLINE, and EMBASE databases were searched to identify relevant articles published until 2020. The patient’s perioperative and oncologic outcomes were analyzed. Statistical analysis was performed using the statistical software RevMan version 5.4.

**Results:**

A total of 17 studies, including four randomized controlled trials, published between 2011 and 2020 were selected. In total, 1,846 patients received low ligation of the IMA plus high dissection of lymph nodes (LL+HD), and 2,648 patients received high ligation of the IMA (HL). LL+HD was associated with low incidence of anastomotic leakage (*p* < 0.001), borderline long operative time (*p* = 0.06), and less yields of total lymph nodes (*p* = 0.03) but equivalent IMA root lymph nodes (*p* = 0.07); moreover, LL+HD exhibited non-inferior long-term oncological outcomes.

**Conclusion:**

In comparison with HL, LL+HD was an effective and safe oncological procedure for sigmoid colon and rectal cancers. Therefore, to ligate the IMA below the level of the left colic artery with D3 high dissection for sigmoid colon and rectal cancers might be suggested once the surgeons are familiar with this technique.

**Systematic Review Registration:**

INPLASY.com, identifier 202190029.

## Introduction

For the optimal surgical treatment of sigmoid colon and rectal cancers, surgeons should accomplish the following: total mesorectal excision, R0 resection, adequate lymph node harvest, adequate distal resection margin (DRM), and negative circumferential resection margin involvement. Furthermore, a secure anastomosis is crucial for good surgical results, and blood supply at the anastomotic site and tension-free anastomosis are particularly essential to prevent anastomotic insufficiency.

Whether high or low ligation of the inferior mesenteric artery (IMA) is optimal for rectal and sigmoid colon cancers is controversial. Surgeons believe that high ligation improves lymph node yield (leading to accurate staging and better prognosis) and complete mobilization to release anastomotic tension. However, high ligation of the IMA (HL) theoretically compromises blood supply to the anastomosis due to removal of the left colic artery (LCA) and raises the concern of increasing the risk of related complications, including bowel ischemia, anastomotic leakage (AL), and anastomosis stenosis. These are particularly common in patients with vascular disease, obesity, or an advanced age with comorbidities.

The benefit of HL in lowering recurrence and prolonging survival was also challenged because the lymph node metastasis rate of the IMA root was relatively low (1). Furthermore, the autonomic nerve plexus is potentially vulnerable during HL and may delay recovery of bowel function and subsequently impair genitourinary function.

Several meta-analyses have compared low and high ligations for superiority in reducing surgical complications and non-inferiority in oncologic outcomes (2–4). However, the studies have been heterogeneous in terms of tumor location, cancer stage, and surgery type. In particular, the level of lymph node clearance has been poorly defined. Some surgeons have performed low ligation of the IMA with lymph node clearance around the IMA root (D3 lymph node dissection) (5–10), whereas others have performed low ligation only and have left apical nodes (station 253) untouched (11, 12). Because D3 lymph node dissection has been non-uniform in the included studies, perioperative and oncological outcomes could not be precisely accessed through a meta-analysis. In this study, we conducted a meta-analysis for comparing high and low ligation of the IMA in surgery for sigmoid colon and rectal cancers, with emphasis on D3 lymph node dissection at the IMA root in all included studies, which were never rigorously studied before.

## Methods

### Study Design

The meta-analysis was conducted and reported according to the Preferred Reporting Items For Systematic Reviews And Meta-Analyses (PRISMA) extension statement. The protocol is registered on INPLASY.com (INPLASY202190029).

### Search Strategies

This meta-analysis was performed in February 2021. We comprehensively searched the PubMed, MEDLINE, and EMBASE databases for articles referring to high and low ligation of the IMA for treating sigmoid colon and rectal cancers. HL denotes that the IMA was ligated at its origin from the abdominal aorta, and low ligation denotes the ligation level was distal to the origin of the LCA. Combinations of the following search terms were used: “sigmoid neoplasm”, “rectal neoplasm”, “left colic artery”, and “inferior mesenteric artery”. The databases were searched for relevant studies from database inception to 2020. After initial screening, two authors independently reviewed and assessed the titles and abstracts of the studies and excluded obviously irrelevant articles. The full texts of the remaining studies were examined to decide their eligibility.

### Inclusion and Exclusion Criteria

The inclusion criteria of our study were as follows: 1) human participants with comparison of high and low ligation of the IMA during curative resection of sigmoid colon or rectal cancer, regardless of the surgical approach (open, laparoscopic, or robotic surgery); and 2) reported at least one of the outcome measures mentioned below. Articles in all languages were eligible for inclusion. In cases of duplicate articles, only the latest published version was included.

The exclusion criteria of this study were as follows: 1) letters, comments, review articles, and case reports; 2) studies without a control group; and 3) surgical procedures involving only low ligation of the IMA without D3 lymph node dissection (high dissection).

### Data Extraction

Two authors (T-CY and H-LT) independently extracted primary relevant data from the studies. The following data were extracted from the included studies: sex, age, the number of patients in each treatment group, tumor location, TNM stage, publication year, country of the study, study type [i.e., non-randomized study vs. randomized controlled trial (RCT)], surgery type (i.e., open, laparoscopic, or robotic surgery), and perioperative and long-term oncological outcomes. Disagreement was resolved through consensus.

Patient’s clinical outcomes were classified according to the following four categories: postoperative morbidity, intraoperative indices, postoperative recovery, and oncologic outcomes including survival and recurrence. Postoperative morbidity outcomes included AL, postoperative ileus, postoperative urinary dysfunction, surgical site infection (SSI), and overall complications. Among them, the AL rate was the primary outcome of the present meta-analysis. Intraoperative indices included intraoperative blood loss, operative time, and conversion rate. Postoperative recovery outcomes included time required for bowel function recovery and hospital length of stay (LOS). Survival and recurrence outcomes included 5-year overall survival (OS) rate and 5-year disease-free survival (DFS) rate for patients at all stages, as well as for stage III patients only. Furthermore, systemic and local recurrence rates were included in this category. Continuous variables from studies reported in median number and interquartile range were not extracted.

### Quality Assessment

The quality and bias risk of the included studies were assessed independently by two authors (T-CY and H-LT); disagreements were settled through discussion. The Newcastle–Ottawa Scale (NOS) was used to assess the quality of non-randomized clinical studies (13). Studies were judged based on patient selection, exposure ascertainment, group comparability, and patient outcomes. The total NOS score ranges from 0 to 9 stars; a score of ≥6 stars indicates high quality. The Jadad scoring system was used to assess the bias risk of RCTs (14). This scoring system is based on three specific items: randomization, blinding, and withdrawals or dropouts. The total score ranges from 0 to 5; a score of ≥3 indicates high-quality evidence.

### Statistical Analysis

Statistical analysis was performed using the statistical software Review Manager (RevMan) Version 5.4 (The Cochrane Collaboration, The Nordic Cochrane Centre, Copenhagen, 2020). The odds ratios (ORs) and mean differences (MDs), with 95% CIs, were calculated for dichotomous and continuous variables, respectively. Heterogeneities were evaluated using χ^2^ and I^2^ tests, with I^2^ results of 25%–50%, 50%–75%, and >75%, considered to indicate low, moderate, and high heterogeneity, respectively (15). Studies with *p* < 0.10 and I^2^ > 25% indicated substantial heterogeneity. If heterogeneity existed with I^2^ > 25%, the random-effects model was used to estimate the pooled OR or MD (DerSimonian and Laird method) (16). Otherwise, the fixed-effects model was adopted (Mantel–Haenszel method or inverse variance method) (17). The Z test (and the related *p*-value) was used to assess the overall effect. Statistical significance was reached at *p* < 0.05. Publication bias was assessed using funnel plots.

## Results

### Study Characteristics

**Figure 1:**
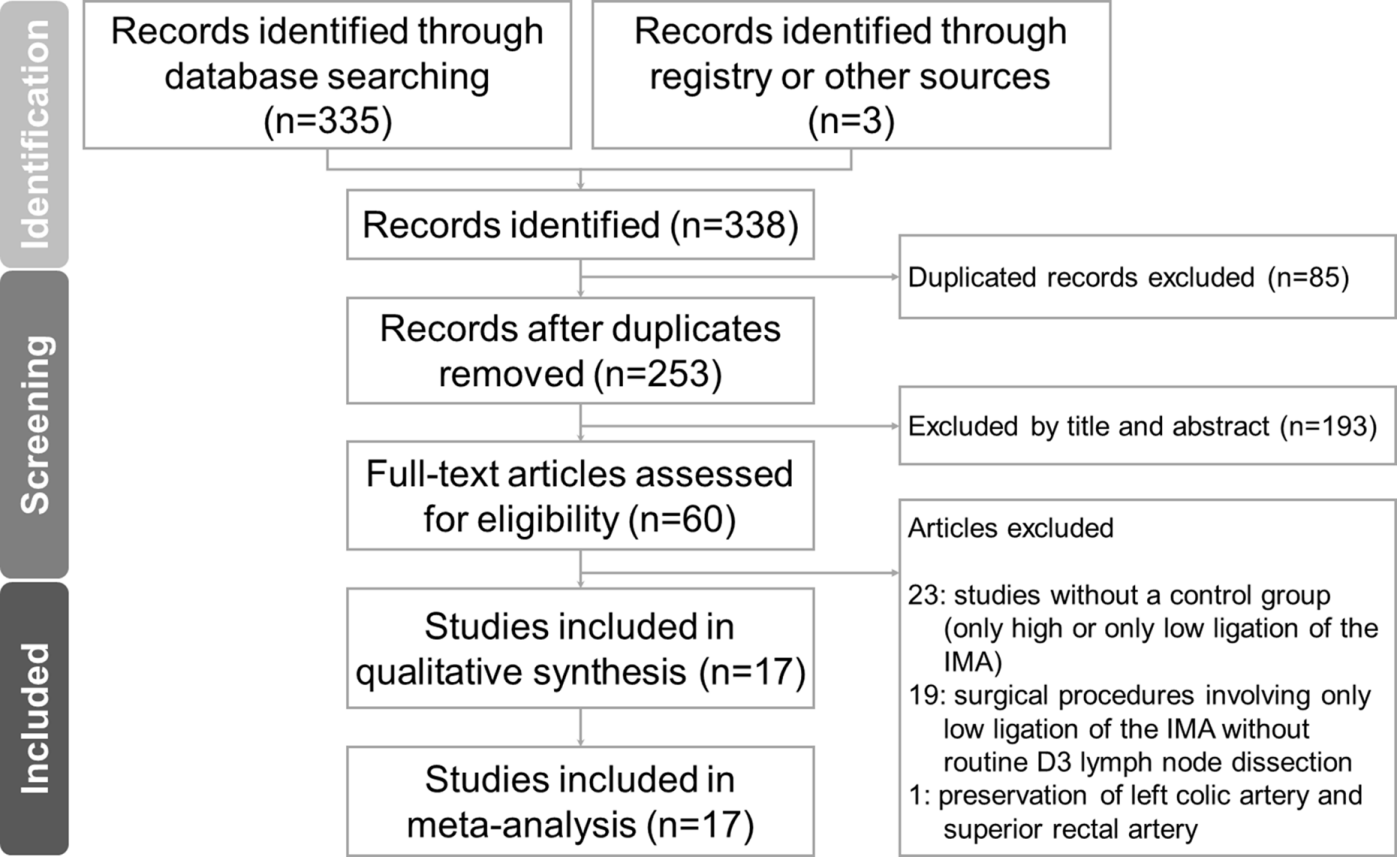
Flow diagram showing the literature search procedure.

**Figure 2:**
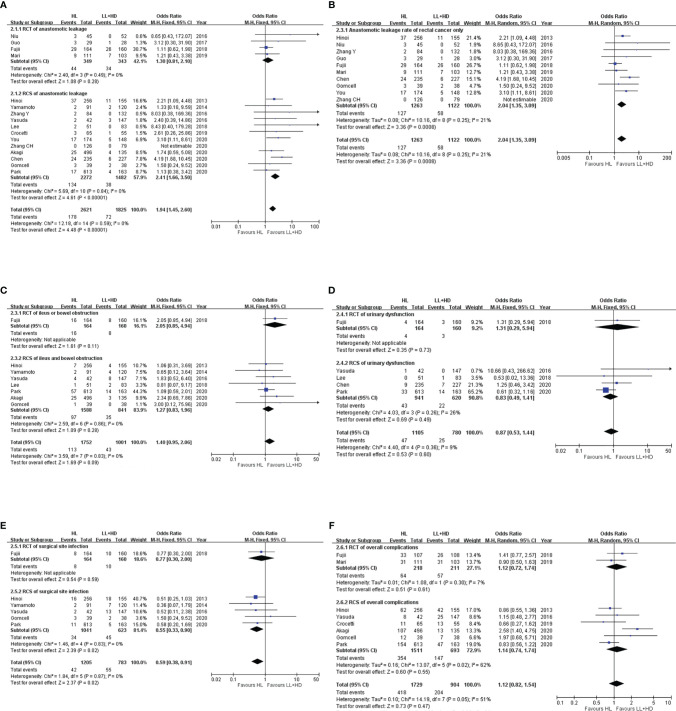
Meta-analysis of postoperative morbidity. **(A)** Forest plot of the anastomotic leakage following HL versus LL+HD. **(B)** Forest plot of anastomotic leakage in rectal cancer following HL versus LL+HD. **(C)** Forest plot of postoperative ileus following HL versus LL+HD. **(D)** Forest plot of urinary dysfunction following HL versus LL+HD. **(E)** Forest plot of the surgical site infection following HL versus LL+HD. **(F)** Forest plot of the total complications following HL versus LL+HD. HL, high ligation of the inferior mesenteric artery; LL+HD, low ligation of the inferior mesenteric artery plus high dissection of lymph nodes.

**Figure 3:**
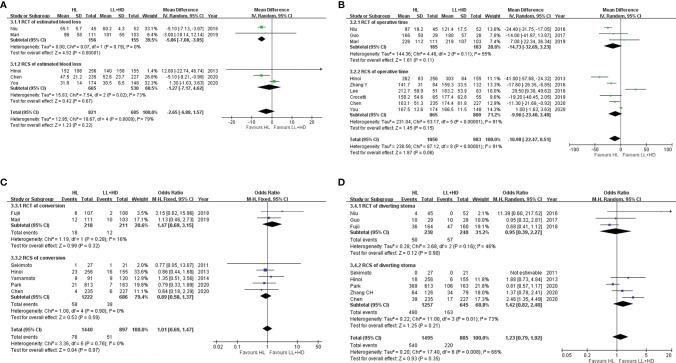
Meta-analysis of intraoperative indices. **(A)** Forest plot of intraoperative blood loss with HL versus LL+HD. **(B)** Forest plot of the operative time with HL versus LL+HD. **(C)** Forest plot of the conversion rate with HL versus LL+HD. **(D)** Forest plot of diverting stoma with HL versus LL+HD. HL, high ligation of the inferior mesenteric artery; LL+HD, low ligation of the inferior mesenteric artery plus high dissection of lymph nodes.

**Figure 4:**
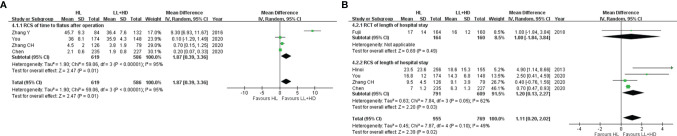
Meta-analysis of postoperative recovery. **(A)** Forest plot of bowel function recovery following HL versus LL+HD. **(B)** Forest plot of the length of hospital stay following HL versus LL+HD. HL, high ligation of the inferior mesenteric artery; LL+HD, low ligation of the inferior mesenteric artery plus high dissection of lymph nodes.

**Figure 5:**
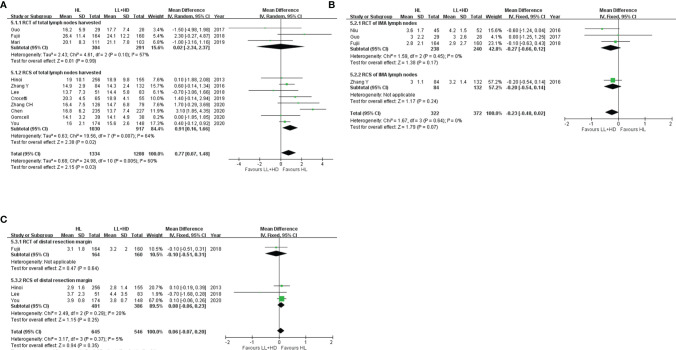
Meta-analysis of surgical quality. **(A)** Forest plot of the total lymph nodes harvested with HL versus LL+HD. **(B)** Forest plot of IMA lymph nodes harvested with HL versus LL+HD. **(C)** Forest plot of the distal resection margin with HL versus LL+HD. HL, high ligation of the inferior mesenteric artery; LL+HD, low ligation of the inferior mesenteric artery plus high dissection of lymph nodes; IMA, inferior mesenteric artery.

**Figure 6:**
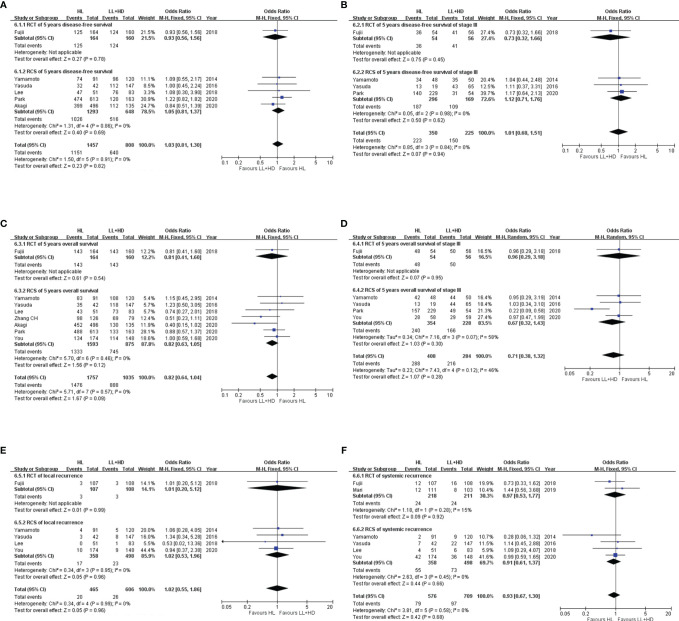
Meta-analysis of survival and recurrence. **(A)** Forest plot of DFS (any stage) following HL versus LL+HD. **(B)** Forest plot of DFS (stage III) following HL versus LL+HD. **(C)** Forest plot of OS (any stage) following HL versus LL+HD. **(D)** Forest plot of OS (stage III) following HL versus LL+HD. **(E)** Forest plot of local recurrence following HL versus LL+HD. **(F)** Forest plot of systemic recurrence following HL versus LL+HD. HL, high ligation of the inferior mesenteric artery; LL+HD, low ligation of the inferior mesenteric artery plus high dissection of lymph nodes; DFS, disease-free survival; OS, overall survival.

**Figure 1** presents the flowchart of the study selection procedure for this meta-analysis. A total of 17 articles published between March 2011 and September 2020 were included in this meta-analysis (5, 6, 8–10, 18–29). Of these, 13 were retrospective cohort studies (RCSs) (6, 8–10, 19, 21, 23–29) and four were RCTs (5, 18, 20, 22). The total number of patients was 4,494, which included 1,846 patients who received low ligation of the IMA plus high dissection of lymph nodes (LL+HD) and 2,648 patients who received HL. The characteristics of the included studies are listed in **Table 1**. The results of the methodological assessment of the included studies using the NOS and Jadad scoring system are shown in **Tables 2**, **3**. All the outcomes are displayed in **Figures 2**–**6**.

**Table 1:** Characteristics of the studies included in this meta-analysis.

Study	Year	Country	Age (mean)	Male (%)	Number of patients		Tumor location	Tumor stage	Type of surgery	Type of study
					HL	LL+HD				
**Sekimoto M (****8****)**	2011	Japan	63.3	23/48 (47.9)	27	21	Sigmoid colon and rectum	NS	Lap.	RCS
**Hinoi T (****6****)**	2013	Japan	62	254/411 (61.8)	256	155	Middle and low rectum	0–IV	Lap.	RCS
**Yamamoto M (****9****)**	2014	Japan	64.6	112/211 (53.1)	91	120	Sigmoid and rectosigmoid colon	II–III	Lap.	RCS
**Niu J (****18****)**	2016	China	50.7	52/97 (53.6)	45	52	Rectum	I–III	Lap.	RCT
**Zhang Y (****19****)**	2016	China	64.7	128/216 (59.3)	84	132	Rectum	NS	Lap	RCS
**Yasuda K (****10****)**	2016	Japan	67.2	118/189 (62.4)	42	147	Sigmoid colon and rectum	I–III	NS	RCS
**Guo Y (****5****)**	2017	China	60.7	33/57 (57.9)	29	28	Rectum	I–III	Lap.	RCT
**Fujii S (****20****)**	2018	Japan	65.8	200/324 (61.7)	164	160	Rectum	0–IV	Lap. and open	RCT
**Lee KH (****21****)**	2018	Korea	66.4	93/134 (69.4)	51	83	Sigmoid colon	I–III	Lap.	RCS
**Mari G (****22****)**	2019	Italy	68	128/214 (59.8)	111	103	Rectum	I–IV	Lap.	RCT
**Crocetti D (****23****)**	2019	Italy	62.8	56/120 (46.7)	65	55	Sigmoid colon and rectum	I–III	Lap.	RCS
**Akagi T (****24****)**	2020	Japan	63.1	379/631 (60.1)	496	135	Sigmoid colon and rectosigmoid	II–III	Lap. and open	RCS
**Gomcell L (****25****)**	2020	Turkey	62.0	46/77 (59.7)	39	38	Rectum	II–III	Robotic	RCS
**Park S (** 26)	2020	Korea	62	513/776 (66.1)	613	163	Sigmoid and rectum	0–IV	Lap.	RCS
**Zhang CH (****27****)**	2020	China	60.7	112/205 (54.6)	126	79	Rectum	I–III	Lap.	RCS
**You X (****28****)**	2020	China	57.6	215/322 (66.8)	174	148	Rectum	0–III	Lap.	RCS
**Chen J (****29****)**	2020	China	58.2	244/462 (52.8)	235	227	Rectum	I–III	Lap.	RCS

HL, high ligation of the inferior mesenteric artery; LL+HD, low ligation of the IMA plus high dissection of lymph nodes; Lap., laparoscopic; RCT, randomized controlled trial; RCS, retrospective cohort study; NS, not stated; IMA, inferior mesenteric artery.

**Table 2:** Bias risk in the randomized controlled trials as assessed by the Jadad scoring system.

Study	Year	Country	Random sequence	Double blind method	Withdrawals and dropouts	Total
**Niu J (****18****)**	2016	China	1	0	1	2
**Guo Y (****5****)**	2017	China	2	1	1	4
**Fujii S (****20****)**	2018	Japan	2	0	1	3
**Mari G (****22****)**	2019	Italy	2	0	0	2

**Table 3:** Quality of non-randomized studies as assessed by the Newcastle–Ottawa Scale.

Study	Year	Country	Selection of the research object	Comparability between groups	Measurement result	Total
**Sekimoto M (****8****)**	2011	Japan	2	1	3	6
**Hinoi T (****6****)**	2013	Japan	4	2	2	8
**Yamamoto M (****9****)**	2014	Japan	2	1	3	6
**Zhang Y (****19****)**	2016	China	4	1	3	8
**Yasuda K (****10****)**	2016	Japan	4	1	3	8
**Lee KH (****21****)**	2018	Korea	2	2	3	7
**Crocetti D (****23****)**	2019	Italy	4	1	3	8
**Akagi T (****24****)**	2020	Japan	4	1	3	8
**Gomcell L (****25****)**	2020	Turkey	4	2	2	8
**Park S (****26****)**	2020	Korea	4	1	2	7
**Zhang CH (****27****)**	2020	China	4	1	3	8
**You X (****28****)**	2020	China	4	2	3	9
**Chen J (****29****)**	2020	China	4	1	3	8

### Meta-Analysis of Postoperative Morbidity

#### Anastomotic Leakage

A total of four RCTs (5, 18, 20, 22) and 12 RCSs (6, 9, 10, 19, 21, 23–29) were included, consisting of 4,446 patients. No heterogeneity existed among the studies. The analysis revealed that the incidence of AL was significantly higher with HL than with LL+HD (OR: 1.94, 95% CI: 1.45–2.60, *p* < 0.001; **Figure 2A**). The AL rate of rectal cancer was also significantly higher with HL than with LL+HD (OR: 2.04, 95% CI: 1.35–3.09, *p* < 0.001; **Figure 2B**).

#### Postoperative Ileus

A total of one RCT (20) and seven RCSs (6, 9, 10, 21, 24–26) were included, involving 2,753 patients. No heterogeneity existed among the studies. The analysis revealed no difference in postoperative ileus incidence between HL and LL+HD (OR: 1.40, 95% CI: 0.95–2.06, *p* = 0.09; **Figure 2C**).

#### Postoperative Urinary Dysfunction

A total of one RCT (20) and four RCSs (10, 21, 26, 29) were included, involving 1,885 patients. No heterogeneity existed among the studies. The analysis revealed no difference in the incidence of postoperative urinary dysfunction between HL and LL+HD (OR: 0.87, 95% CI: 0.49–1.41, *p* = 0.60; **Figure 2D**).

#### Surgical Site Infection

A total of one RCT (20) and five RCSs (6, 9, 10, 25, 26) were included, involving 1,988 patients. No heterogeneity existed among the studies. The analysis revealed that the incidence of SSI was significantly lower with HL than with LL+HD (OR: 0.59, 95% CI: 0.38–0.91, *p* = 0.02; **Figure 2E**).

#### Overall Complications

A total of two RCTs (20, 22) and six RCSs (6, 10, 23–26) were included, involving 2,633 patients. Heterogeneity among the studies was moderate. The analysis revealed no difference in the overall complication rate between HL and LL+HD (OR: 1.12, 95% CI: 0.82–1.54, *p* = 0.47; **Figure 2F**).

### Meta-Analysis of Intraoperative Indices

#### Blood Loss

A total of two RCTs (18, 22) and three RCSs (6, 28, 29) were included, involving 1,506 patients. Heterogeneity among the studies was high. The analysis revealed no difference in the estimated blood loss between HL and LL+HD (MD: −2.65, 95% CI: −6.88 to 1.57, *p* = 0.22; **Figure 3A**).

#### Operative Time

A total of three RCTs (5, 18, 22) and six RCSs (6, 19, 21, 23, 28, 29) were included, involving 2,033 patients. Heterogeneity among the studies was high. The analysis revealed that the operation time of HL was significantly borderline shorter than that of LL+HD (MD: −10.98, 95% CI: −22.47 to 0.51, *p* = 0.06; **Figure 3B**).

#### Conversion Rate

A total of two RCTs (20, 22) and five RCSs (6, 8, 9, 26, 29) were included, involving 2,337 patients. No heterogeneity existed among the studies. The analysis revealed no difference in the conversion rate between HL and LL+HD (OR: 1.01; 95% CI: 0.69–1.47; *p* = 0.97; **Figure 3C**).

#### Diverting Stoma

A total of three RCTs (5, 18, 20) and five RCSs (6, 8, 26, 27, 29) were included, involving 2,380 patients. Heterogeneity among the studies was moderate. The analysis revealed no difference in the incidence of diverting stoma between HL and LL+HD (OR: 1.23, 95% CI: 0.79–1.92, *p* = 0.35; **Figure 3D**).

### Meta-Analysis of Postoperative Recovery

#### Time of Bowel Function Recovery

A total of four RCSs (19, 27–29) were included, involving 1,205 patients. Heterogeneity among the studies was high. The analysis revealed that the time of bowel function recovery of HL was significantly longer than that of LL+HD (MD: 1.87, 95% CI: 0.39–3.36, *p* = 0.01; **Figure 4A**).

#### Length of Hospital Stay

A total of one RCT (20) and four RCSs (6, 27–29) were included, involving 1,724 patients. Heterogeneity among the studies was low. The analysis revealed that the LOS was significantly longer with HL than with LL+HD (MD: 1.11, 95% CI: 0.20–2.02, *p* = 0.02; **Figure 4B**).

### Meta-Analysis of Surgical Quality

#### Total Lymph Nodes Harvested

A total of three RCTs (5, 20, 22) and eight RCSs (6, 19, 21, 23, 25, 27–29) were included, involving 2,542 patients. Heterogeneity among the studies was moderate. The analysis revealed significant more total lymph nodes harvested with HL than with LL+HD (MD: 0.77, 95% CI: 0.07 to 1.48, *p* = 0.03; **Figure 5A**).

#### Inferior Mesenteric Artery Root Lymph Nodes Harvested

A total of three RCTs (5, 18, 20) and one RCS (19) were included, involving 694 patients. No heterogeneity existed among the studies. The analysis revealed no difference in the IMA root lymph nodes harvested between HL and LL+HD (MD: −0.23, 95% CI: −0.48 to 0.02, *p* = 0.07; **Figure 5B**).

#### Distance of Distal Resection Margin

A total of one RCT (20) and three RCSs (6, 21, 28) were included, involving 1,191 patients. No heterogeneity existed among the studies. The analysis revealed no difference in the distance of DRM between HL and LL+HD (MD: 0.06, 95% CI: −0.07 to 0.20, *p* = 0.35; **Figure 5C**).

### Meta-Analysis of Survival and Recurrence

#### Disease-Free Survival in Patients at All Stages

A total of one RCT (20) and five RCSs (9, 10, 21, 24, 26) were included, involving 2,265 patients. No heterogeneity existed among the studies. The analysis revealed no difference in the DFS between HL and LL+HD (OR: 1.03, 95% CI: 0.81–1.30, *p* = 0.82; **Figure 6A**).

#### Disease-Free Survival at Stage III Disease

A total of one RCT (20) and three RCSs (9, 10, 26) were included, involving 575 patients. No heterogeneity existed among the studies. The analysis revealed no difference in the DFS at stage III disease between HL and LL+HD (OR: 1.01, 95% CI: 0.68–1.51, *p* = 0.94; **Figure 6B**).

#### Overall Survival in Patients at All Stages

A total of one RCT (20) and seven RCSs (9, 10, 21, 24, 26–28) were included, involving 2,792 patients. No heterogeneity existed among the studies. The analysis revealed no difference in OS between HL and LL+HD (OR: 0.82, 95% CI: 0.64–1.04, *p* = 0.09; **Figure 6C**).

#### Overall Survival at Stage III Disease

A total of one RCT (20) and four RCSs (9, 10, 26, 28) were included, involving 692 patients. Heterogeneity among the studies was low. The analysis revealed no difference in OS at stage III disease between HL and LL+HD (OR: 0.71, 95% CI: 0.38–1.32, *p* = 0.28; **Figure 6D**).

#### Local Recurrence

A total of one RCT (20) and four RCSs (9, 10, 21, 28) were included, involving 1,071 patients. No heterogeneity existed among the studies. The analysis revealed no difference in the local recurrence rate between HL and LL+HD (OR: 1.02, 95% CI: 0.55–1.86, *p* = 0.96; **Figure 6E**).

#### Systemic Recurrence

A total of two RCTs (20, 22) and four RCSs (9, 10, 21, 28) were included, involving 1,285 patients. No heterogeneity existed among the studies. The analysis revealed no difference in the systemic recurrence rate between HL and LL+HD (OR: 0.93, 95% CI: 0.67–1.30, *p* = 0.68; **Figure 6F**).

### Publication Bias

**Figure 7:**
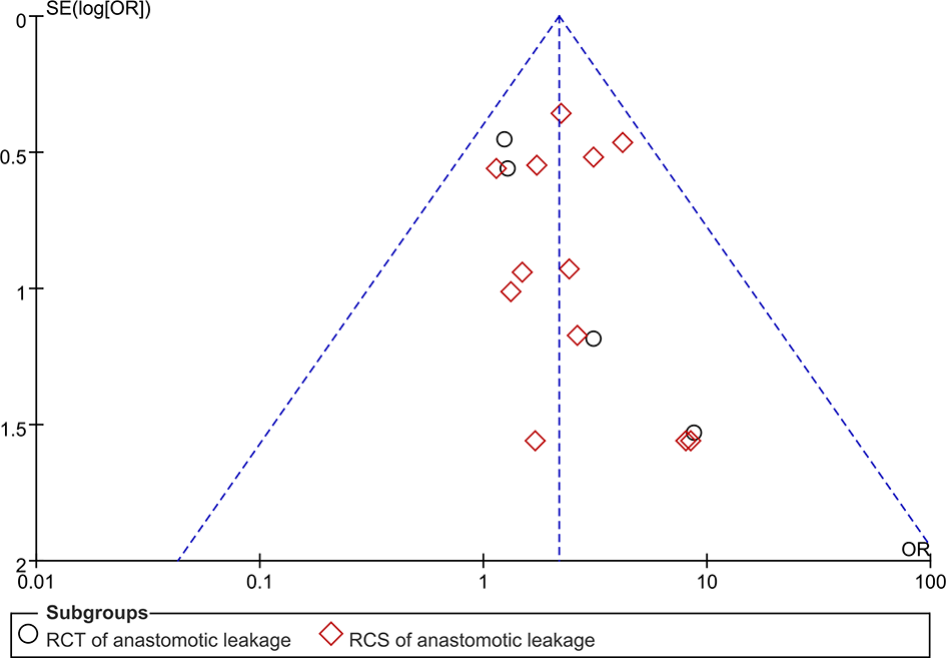
Funnel plot of anastomotic leakage.

Funnel plot analysis was performed on studies assessing AL (**Figure 7**). The ORs of all the studies were within the pooled 95% CIs. In addition, the studies were equally distributed on both sides of the vertical line. This indicated that publication bias was low in our meta-analysis.

### Sensitivity Analysis

We excluded the studies with low Jadad score and recalculated the pooled OR for the primary end point (AL) in the remaining studies. However, the risk of AL remained higher in HL patients (OR: 1.97, 95% CI: 1.45–2.68, *p* < 0.001; **Supplement 1**).

## Discussion

The pooled AL rate after surgery for rectal and sigmoid colon cancers in the current meta-analysis was 2.5% in patients who received LL+HD and 6.5% in patients who received the conventional HL. AL incidence was significantly reduced with LL+HD compared with the standard HL procedure (OR: 1.94, 95% CI: 1.45–2.60, *p* < 0.001). The inconsistency between the RCTs and the RCSs might because the case number of the RCTs was relatively small compared with that of the RCSs, which made the statistical significance hard to reach. However, there was still the tendency of a lower AL rate in LL+HD patients in RCTs. Besides, a high and similar AL rate in both HL and LL+HD group was found in one of the included RCTs (20) and was highly weighted in our analysis.

The leakage rate of colorectal or coloanal anastomosis ranged widely; an AL rate of 3%–6% was considered acceptable by well-experienced surgeons (30). Factors influencing AL were complex. Some of them were non-adjustable and were related to patients (e.g., male sex, diabetes, renal insufficiency, obesity, and malnutrition) and tumors (e.g., distal location, bulky, and advanced stage), whereas others were related to preoperative treatment such as preoperative radiotherapy or anti-vascular endothelial growth factor monoclonal antibody treatment (31). The vascular ligation level has frequently been mentioned as a factor of AL because it potentially compromises blood supply to the proximal limb of anastomosis in rectal surgery. Although marginal artery universally consists of intermesenteric connections between the superior mesenteric artery and IMA and offers considerable collateral circulation of the bowel, marginal artery continuity might be interrupted in 5%–7% of individuals at Griffith’s point (32). LCA preservation in these patients is particularly important. Seike et al. used laser Doppler and detected 37%–40% reduction in the blood flow at the proximal site of the anastomosis while the IMA was clamped (33). Two-thirds of individuals aged >65 years received their blood flow beyond the splenic flexure to transverse colon from the IMA according to a digital substrate angiogram study by Zhang et al. (34) Another study using CT angiography demonstrated that the LCA dominated blood supply to the splenic flexure in more than half of the individuals (35). By contrast, Rutegard et al. found no statistically significant difference in leakage rate associated with LCA preservation (36).

Contrary to the National Comprehensive Cancer Network, which emphasizes the number of lymph nodes dissected, lymph node location was also important and affected survival significantly (37). Patients with colorectal cancer (CRC) with apical lymph node metastasis had worse survival and higher incidence of distant metastasis as compared with those without (38–40). Some surgeons insist on D3 lymph node dissection because R0 resection significantly improves recurrence-free survival even in rectal cancer patients with IMA lymph node metastasis as compared with R1 and R2 resection (41). On the other hand, Uehara et al. found that D3 lymph node dissection offered limited benefit to patients with stage III rectal cancer and apical node metastasis (42). The key factor seemed to be the incidence of IMA lymph node metastases, and most of the studies revealed relatively low prevalence, ranging from 1.7% to 3.1% (39, 41, 42). High prevalence of apical node metastasis could still be found in some studies, which made IMA lymph nodes unneglectable (40, 43). In the present analysis, LL+HD yielded less total lymph nodes (*p* = 0.03) but an equivalent amount of IMA lymph nodes compared with HL in surgery for sigmoid colon and rectal cancers (*p* = 0.07). Furthermore, no significant difference existed in total lymph nodes harvested (MD: 0.69, 95% CI: −0.14 to 1.52, *p* = 0.10; **Supplement 2**) in trials with their inferior mesenteric vein ligation level clearly defined (5, 6, 19, 22, 25, 27–29), implying the discrepancy in area of lymphatic clearance and its impact. Oncologic outcome of LL+HD was non-inferior to that of HL in terms of both DFS and OS at any stage of CRC (*p* = 0.82 and 0.09). Even in stage III CRC, this novel technique provided long-term survival benefit to patients (*p* = 0.94 and 0.28). Both local recurrence and systemic recurrence following LL+HD were comparable with those following HL (*p* = 0.96 and 0.68).

Traditionally, the IMA is ligated and transected at the point where it branches off from the abdominal aorta during anterior resection or low anterior resection. This procedure (HL) eliminates the N3 lymph nodes at the IMA root, which is beneficial for radical lymphatic clearance. Furthermore, HL releases the tension of mesentery and contributes to a tension-free anastomosis. Splenic flexure is usually inevitably mobilized when the LCA is sacrificed. However, the position of splenic flexure is sometimes deep or high in the left upper quadrant, making mobilization rather difficult (42). LCA preservation theoretically provides burst blood and allows precise resection to avoid tension at the anastomosis. Splenic flexure mobilization could be omitted during anterior resection or low anterior resection without tension at the anastomotic or risk of anastomotic insufficiency, particularly in patients with a long sigmoid colon (42). The specimen length, particularly the distance of DRM, without splenic flexure mobilization should not be a concern because no difference in DRM was observed following HL and LL+HD as shown in the present analysis (*p* = 0.20).

Low ligation of the IMA was first described in 1908 when abdominal perineal resection was performed for rectal cancer (44). Furthermore, this technique was performed in diverticular disease and reduced the AL rate by three-fourths (45). In CRC management, several meta-analyses have demonstrated that low ligation of the IMA is associated with a low AL rate (2, 46, 47), equivalent harvesting of lymph nodes (2–4, 46, 47), identical recurrence and survival rates (2, 3, 46, 47), similar intraoperative blood loss (2, 4), and borderline increase in operative time (2). Furthermore, few minor analyses have revealed the lower incidence of postoperative urinary dysfunction and the lower need for neostomy (47). However, heterogeneities existed among included studies in terms of tumor location, cancer stage, and surgery type. The level of lymph nodes clearance was particularly poorly defined, with most of these meta-analyses including studies on LL without D3 lymph node dissection. The present analysis compared high and low ligation of the IMA in surgery for sigmoid and rectal cancers, with emphasis on D3 dissection at the IMA root in all included studies. In addition to lower incidences of AL and anastomotic stenosis aforementioned, the meta-analysis revealed no difference in the incidence of postoperative ileus and postoperative urinary dysfunction between HL and LL+HD (*p* = 0.09 and 0.60). The incidence of SSI was higher with LL+HD (*p* = 0.02) and might be due to longer operative time. Overall complication rates were similar in both techniques (*p* = 0.47). Considering intraoperative indices and safety, LL+HD required borderline longer operative time than the standard HL (*p* = 0.06), although high heterogeneity of included studies did exist. Intraoperative blood loss was equivalent between LL+HD and HL regardless of the surgical approach (*p* = 0.22). The necessity of diverting stoma with LL+HD and HL (*p* = 0.35) and the conversion rate was also nearly identical (*p* = 0.97). Bowel function recovered significantly faster after LL+HD in surgery for sigmoid colon and rectal cancers (*p* = 0.01). LOS was significantly shorter with LL+HD than traditional HL as expected (*p* = 0.02). The results of the meta-analysis regarding surgical quality and oncologic outcome were as stated above.

The physiologic urinary function depended largely on the coordination of parasympathetic and sympathetic systems to control bladder emptying and continence. Furthermore, the autonomic nervous system plays a critical role in sexual function for erection and ejaculation in men and pareunia in women. The superior hypogastric plexus (SHP) was potentially vulnerable during the very beginning of the procedure (presacral fascia dissection) and during the vascular approach of high dissection close to the origin of the IMA. Postoperatively, voiding function after removal of the Foley catheter was good in 85% patients following D3 lymph node dissection with the preservation of the LCA and autonomic nerve plexus (48). Additionally, patients who received LL+HD were reported to have better continence, less obstructive urinary symptoms, and better sexual function than those receiving HL; and they had returned to their preoperative levels 9 months after surgery. Moreover, these were evident in objective measurements obtained through uroflowmetric examination and ultrasound (22). Notably, in these studies, surgical techniques of “preservation of autonomous nerve plexus encircling the IMA” and “dissection of apical lymph node IMA without reaching the aortic plane” were particularly highlighted to avoid plexus injury and subsequent impairment in the genitourinary function of patients. Actually, the possibility of iatrogenic injury to the SHP might be equal in extensive lymphatic clearance between high and low ligation of the IMA, unless surgeons (and available studies) paid special attention. A lack of standardized procedures for the skeletonization of the IMA and lymphadenectomy around the IMA root might be the reason for the finding of non-superiority of urinary function after LL+HD compared with HL and failure to recommend one approach over the other in the current meta-analysis (*p* = 0.60).

Bertrand et al. considered that low ligation of the IMA was not sufficiently reproducible to be a standard surgical procedure for CRC due to variation in the division branches of the IMA (49). However, the anatomic variation of the IMA has been well studied, recognized, and categorized into four main types (34, 50). Familiarization with variations in branches was fundamental to the low ligation of the IMA. Despite the high dissection of lymph nodes at the IMA root, the level of inferior mesenteric vein ligation, the decision of splenic flexure mobilization, temporary stoma formation, and even the area of lymphatic clearance were discrepant in the literature review and are not conclusive yet (7–10, 48, 51–56).

Although the rate of IMA root lymph node metastasis was low in the reviewed literature, whether clearance was achieved remains questionable. HL might compromise blood flow to the anastomosis and increase the risk of AL and stenosis. LL+HD reduced the incidence of anastomotic insufficiency and yielded as many IMA root lymph nodes as HL did. Survival and recurrence were non-inferior with LL+HD compared with standard HD for CRC surgery. Despite a borderline longer operative time, patients who received the novel technique recovered faster than those who received the traditional procedure, regardless of the surgical approach. IMA ligation below the LCA level with D3 high dissection would be the preferred technique during sigmoid colon and rectal cancer surgeries when surgeons were familiar with this operative technique. Limitations still existed in the current meta-analysis. First, some important outcomes were reported in minor studies (≤4 studies and included RCS only). Second, moderate-to-high heterogeneities among included studies on several specific outcomes were insurmountable in this meta-analysis. Third, surgical details, particularly the extent of D3 lymphatic clearance and the method of preserving the SHP, are not standardized yet. More comprehensive and updated searching of databases should be conducted in the future.

## Conclusion

LL+HD is an effective and safe procedure for treating sigmoid colon and rectal cancers. It reduces the incidence of anastomotic insufficiency, which is the most severe complication following colorectal surgery. Furthermore, it harvested equivalent IMA root lymph nodes as HL. For CRC surgery, survival and recurrence are non-inferior with LL+HD compared with standard HL.

## Data Availability Statement

The original contributions presented in the study are included in the article/**Supplementary Material**. Further inquiries can be directed to the corresponding authors.

## Funding

This work was supported by grants through funding from the Ministry of Science and Technology (MOST 109-2314-B-037-035, MOST 109-2314-B-037-040, MOST 109-2314-B-037-046-MY3) and the Ministry of Health and Welfare (MOHW109-TDU-B-212-134026, MOHW109-TDU-B-212-114006, MOHW110-TDU-B-212-1140026) and funded by the health and welfare surcharge of on tobacco products, and the Kaohsiung Medical University Hospital (KMUH109-9R32, KMUH109-9R33, KMUH109-9R34, KMUH109-9M30, KMUH109-9M31, KMUH109-9M32, KMUH109-9M33, KMUHS10903, KMUHSA10903, KMUH-DK(C)110010, KMUH-DK(B)110004-3) and KMU Center for Cancer Research (KMU-TC109A04-1) as well as and a KMU Center for Liquid Biopsy and Cohort Research Center Grant (KMU-TC109B05), Kaohsiung Medical University. In addition, this study was supported by the Grant of Taiwan Precision Medicine Initiative, Academia Sinica, Taiwan, R.O.C.

## Conflict of Interest

The authors declare that the research was conducted in the absence of any commercial or financial relationships that could be construed as a potential conflict of interest.

## Publisher’s Note

All claims expressed in this article are solely those of the authors and do not necessarily represent those of their affiliated organizations, or those of the publisher, the editors and the reviewers. Any product that may be evaluated in this article, or claim that may be made by its manufacturer, is not guaranteed or endorsed by the publisher.
