# Supplementary material for: Low Ligation Plus High Dissection Versus High Ligation of the Inferior Mesenteric Artery in Sigmoid Colon and Rectal Cancer Surgery: A Meta-Analysis
Source: Front Oncol. 2021 Nov 11;11:774782. doi: 10.3389/fonc.2021.774782 (PMC8632045; doi:10.3389/fonc.2021.774782)
Supplement: Supplementary file 1 [file DataSheet_1.zip › Supplement 3.DOCX]

**Supplement 3. Articles excluded due to their surgical procedures involving low ligation without statements of routine D3 lymph node dissection**

| **Title** | **Authors** | **Publication year** |
| --- | --- | --- |
| Survival after high or low ligation of the inferior mesenteric artery during curative surgery for rectal cancer | Pezim ME, Nicholls RJ. | 1984 |
| High versus low ligation of the inferior mesenteric artery in rectal cancer | Surtees P, Ritchie JK, Phillips RK. | 1990 |
| Flush aortic tie versus selective preservation of the ascending left colic artery in low anterior resection for rectal carcinoma | Corder AP, Karanjia ND, Williams JD, Heald RJ. | 1992 |
| Proximal bowel necrosis after high ligation of the inferior mesenteric artery in colorectal surgery. | Tsujinaka S; Kawamura YJ; Tan KY; Mizokami K; Sasaki J; Maeda T; Kuwahara Y; Konishi F; Lefor A | 2012 |
| High tie in anterior resection for rectal cancer confers no increased risk of anastomotic leakage. | Rutegård M; Hemmingsson O; Matthiessen P; Rutegård J | 2012 |
| High tie versus low tie vascular ligation of the inferior mesenteric artery in colorectal cancer surgery: impact on the gain in colon length and implications on the feasibility of anastomoses. | Bonnet S; Berger A; Hentati N; Abid B; Chevallier JM; Wind P; Delmas V; Douard R | 2012 |
| Randomized clinical trial of defaecatory function after anterior resection for rectal cancer with high versus low ligation of the inferior mesenteric artery | Matsuda K, Hotta T, Takifuji K, Yokoyama S, Oku Y, Watanabe T, Mitani Y, Ieda J, Mizumoto Y, Yamaue H. | 2015 |
| Comparison of laparoscopic sigmoidectomy with and without preservation of the superior rectal artery: a single-institution retrospective study | Wakahara T, Toyokawa A, Ashitani H, Tsuchida S, Hasegawa Y. | 2015 |
| [Effect of ligation level of inferior mesenteric artery on postoperative defecation function in patients with rectal cancer] | Wang Q, Zhang C, Zhang H, Wang Y, Yuan Z, Di C. | 2015 |
| High arterial ligation and risk of anastomotic leakage in anterior resection for rectal cancer in patients with increased cardiovascular risk. | Boström P; Haapamäki MM; Matthiessen P; Ljung R; Rutegård J; Rutegård M | 2015 |
| Level of vascular tie and its effect on functional outcome 2 years after anterior resection for rectal cancer | Kverneng Hultberg D, Afshar AA, Rutegård J, Lange M, Haapamäki MM, Matthiessen P, Rutegård M. | 2017 |
| Oncological Outcomes following Rectal Cancer Surgery with High or Low Ligation of the Inferior Mesenteric Artery. | Matsuda K; Yokoyama S; Hotta T; Takifuji K; Watanabe T; Tamura K; Mitani Y; Iwamoto H; Mizumoto Y; Yamaue H | 2017 |
| High versus low ligation of inferior mesenteric vessels in rectal cancer surgery: A retrospective cohort study | Dimitriou N, Felekouras E, Karavokyros I, Pikoulis E, Vergadis C, Nonni A, Griniatsos J. | 2018 |
| Low Tie Compared to High Tie Vascular Ligation of the Inferior Mesenteric Artery in Rectal Cancer Surgery Decreases Postoperative Complications Without Affecting Overall Survival | Nayeri M, Iskander O, Tabchouri N, Artus A, Michot N, Muller O, Giger-Pabst U, Bourlier P, Kraemer-Bucur A, Lecomte T, Salame E, Ouaissi M. | 2019 |
| High Tie or not in Resection for Cancer in the Sigmoid Colon? | Olofsson F, Buchwald P, Elmståhl S, Syk I. | 2019 |
| [Effect of preservation of left colic artery on postoperative anastomotic leakage of patients with rectal cancer after neoadjuvant therapy] | Teng WH, Wei C, Liu WJ, Liu S, Chen S, Zang WD. | 2019 |
| Association of high ligation versus low ligation of the inferior mesenteric artery on anastomotic leak, postoperative complications, and mortality after minimally invasive surgery for distal sigmoid and rectal cancer | Draginov A, Chesney TR, Quereshy HA, Chadi SA, Quereshy FA. | 2020 |
| Is Low Inferior Mesenteric Artery Ligation Worthwhile to Prevent Urinary and Sexual Dysfunction After Total Mesorectal Excision for Rectal Cancer? | Fiori E; Crocetti D; Lamazza A; DE Felice F; Sterpetti AV; Irace L; Mingoli A; Sapienza P; DE Toma G | 2020 |
| Preservation of the Arterial Arc Formed by Left Colic Artery, Proximal Inferior Mesenteric Artery, and the First Branch of Sigmoid Arteries in Anus Saving Treatment of Low Rectal Cancer. | Shaibu Z; Chen ZH; Theophilus A; Mzee SAS | 2020 |
