# Supplementary figures and images for: Low Ligation Plus High Dissection Versus High Ligation of the Inferior Mesenteric Artery in Sigmoid Colon and Rectal Cancer Surgery: A Meta-Analysis
Source: Front Oncol. 2021 Nov 11;11:774782. doi: 10.3389/fonc.2021.774782 (PMC8632045; doi:10.3389/fonc.2021.774782)

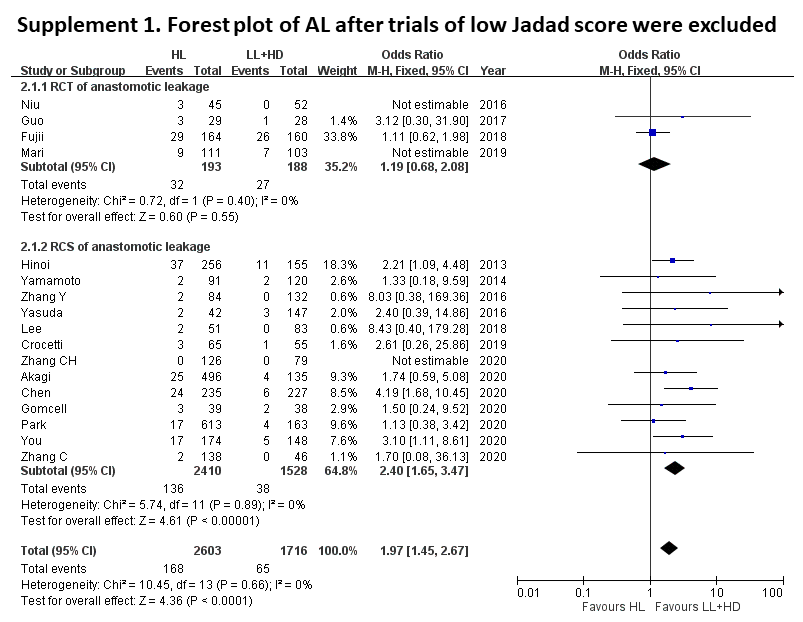

Supplement: Supplementary file 1 [file DataSheet_1.zip › Supplement 1.TIF]

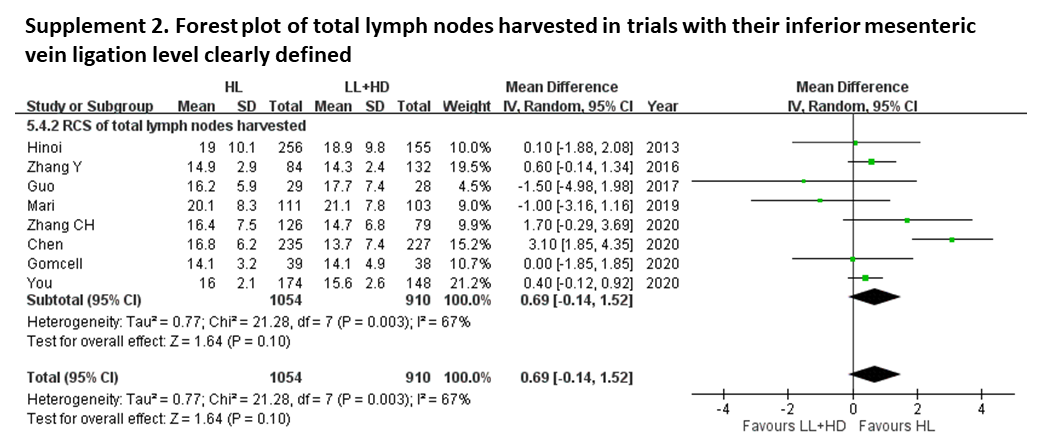

Supplement: Supplementary file 1 [file DataSheet_1.zip › Supplement 2.TIF]
